# The prognostic value of the tertiary lymphoid structure in gastrointestinal cancers

**DOI:** 10.3389/fimmu.2023.1256355

**Published:** 2023-10-06

**Authors:** Aoyang Yu, Menghan Cao, Kaile Zhang, Yule Yang, Luyao Ma, Xinran Zhang, Yang Zhao, Xiao Ma, Zhixiang Fan, Zhengxiang Han, Hongmei Wang

**Affiliations:** Department of Oncology, The Affiliated Hospital of Xuzhou Medical University, Jiangsu, China

**Keywords:** tertiary lymphoid structure (TLS), gastrointestinal (GI) cancers, meta-analysis, prognosis, biomarkers

## Abstract

**Background:**

Numerous studies and research papers have provided evidence suggesting that tertiary lymphoid structures (TLS) play a crucial role in combating and suppressing tumor growth and progression. Despite the wealth of information on the significance of TLS in various types of cancer, their prognostic value in gastrointestinal (GI) cancers remains uncertain. Therefore, this meta-analysis investigated the prognostic value of TLS in GI cancers.

**Methods:**

We searched Web of science, Pubmed, Embase and Cochrane Library for studies that met the requirements as of May 1, 2023, and the hazard ratio (HR) and the corresponding 95% confidence interval (CI) were included in the analysis. The bioinformatics analysis results based on the TCGA database are used to supplement our research.

**Results:**

The meta-analysis included 32 studies involving 5778 patients. The results of comprehensive analysis showed that TLS-High is associated with prolonged OS (HR=0.525,95%CI:0.447-0.616 (P < 0.001), RFS (HR=0.546,95%CI:0.461-0.647, P < 0.001), DFS (HR=0.519,95%CI:0.417-0.646, P < 0.001) and PFS (HR=0.588,95%CI:0.406-0.852, P=0.005) in GI cancer. Among the patients who received immunotherapy, TLS-High is associated with significantly prolonged OS (HR=0.475, 95%CI:0.282-0.799, P=0.005) and PFS(HR=0.576, 95%CI:0.381-0.871, P=0.009). It is worth noting that subgroup analysis showed that there was no significant relationship between TLS and OS(HR=0.775, 95%CI:0.570-1.053,P=0.103) in CRC. And when Present is used as the cut-off criteria of TLS, there is no significant correlation between TLS and OS (HR=0.850, 95%CI:0.721-1.002, P=0.053)in HCC.

**Conclusion:**

TLS is a significant predictor of the prognosis of GI cancers and has the potential to become a prognostic biomarker of immunotherapy-related patients.

**Systematic review registration:**

https://www.crd.york.ac.uk/PROSPERO/#recordDetails, identifier CRD42023443562.

## Introduction

Gastrointestinal (GI) cancers, including esophageal, gastric, liver, biliary system, pancreatic, and colorectal tumors, account for more than a quarter of all cancer incidences worldwide. These types of cancers are responsible for one-third of all cancer-related deaths (1). Some previous studies have indicated that there is a correlation between the occurrence of GI cancers and factors such as smoking, diet, and potential pathogens like EBV (Epstein-Barr virus) and Escherichia coli that produces colibactin (2–5). These factors are linked to the escalating burden of GI cancers. In the past decade, immunotherapy such as anti-PD-1/PD-L1 has greatly improved the prognosis of cancer patients. However, this efficacy is largely limited to patients who have high microsatellite instability (MSI-H) or positive PD-L1 expression. Patients with GI cancers have a relatively low overall response rate to current immunotherapy, and the existing prognostic markers are insufficient to determine which patients can benefit from immunotherapy (6, 7). In addition, the heterogeneity of GI cancers has led to a wide range of clinical, pathological, and molecular characteristics. This diversity poses greater challenges in personalized diagnosis and treatment (8). TLS are formed as ectopic lymph node-like structures within non-lymphoid tissues. Typically, TLS consists of T cells, B cells, fibroblastic reticular cells (FRC) network, high endothelial venules (HEV), and follicular dendritic cells (FDC) (9). Recent literature suggests that the presence of TLS is associated with the prognosis of various gastrointestinal (GI) cancers (10–13). However, there is currently no unified way to evaluate TLS. Some studies classify TLS as positive or negative based on density (11, 14, 15), while other studies consider the presence or absence of TLS as a criterion for evaluation (14, 16). Furthermore, some studies assess the maturity level of TLS (17). These different grouping approaches based on TLS may impact the predictive value of TLS for prognosis. In addition, it has been observed that TLS (Tumor-Localized Immune Response) is not correlated with patient prognosis in certain advanced colorectal cancer cohorts (14, 18). Due to the existence of these controversial conclusions, it is necessary to conduct an analysis to elucidate the role of TLS in GI cancer under different grouping methods. This study performed a systematic review and meta-analysis on the relationship between TLS and the survival outcomes of patients with GI cancer.

## Methods

### Literature search strategies

The meta-analysis was designed and conducted based on the Preferred Reporting Items for Systematic Reviews and Meta-Analyses (PRISMA) reporting guidelines, which are considered the gold standard for reporting systematic reviews and meta-analyses. The specific search strategy is Tertiary Lymphoid Structures OR Lymphoid Structure, Tertiary OR Lymphoid Structures, Tertiary OR Tertiary Lymphoid Structure OR Ectopic Lymphoid-Like Structures OR Lymphoid-Like Structure, Ectopic OR Ectopic Lymphoid Tissues. The protocol for this meta-analysis study can be found in PROSPERO (19).

### Inclusion and exclusion criteria

The eligible studies should meet the following criteria (1): GI cancers confirmed by pathological diagnosis (2); Detection of the expression levels of TLS in human tumor tissues (3); Providing survival data, including hazard ratio (HR) and 95% confidence interval (CI) measurements for OS, RFS, PFS, or PFS, or providing Kaplan-Meier curves based on TLS grouping (4); Providing the methods for TLS detection and evaluation. The following studies have been excluded from consideration due to various reasons (1): Comment, animal studies, letter, edit, reviews and meta-analysis (2); Conducting multiple studies using the same set of samples or participants (3); No insufficient data or no prognostic information.

### Data extraction and quality assessment

Extract the following data from the included studies: year of publication, region, sample size, sex, cancer type, TLS cut-off criteria, follow-up time(months), survival analysis (OS, RFS, DFS or PFS), HR and 95%CI. If HR and 95% CI are not provided, Engauge Digitizer software version 4.1 was used to plot the Kaplan–Meier curves and extract the multiple survival rates to estimate the HRs and 95% CIs (20). Quality assessment was performed using the Newcastle–Ottawa quality assessment scale (NOS). NOS criteria scores range from 0 (lowest) to 9 (highest), and a NOS score ≥6 is considered a high-quality study. Two reviewers (Kaile Zhang and Yule Yang) independently assessed the quality of the eligible studies and extracted the data, and any disagreement was resolved through discussion with the third (Menghan Cao) (21).

### Bioinformatics analysis

In this study, the gene expression and clinical information of gastrointestinal cancer patients were downloaded from the TCGA database (https://portal.gdc.cancer.gov/). The patients were divided into two groups based on the scores of 9 TLS-related genes (CCR6, CD1D, CD79B, CETP, EIF1AY, LAT, PTGDS, RBP5, and SKAP1), namely the TLSscore high group (upper tertile) and the TLSscore low group (lower tertile) (22). The ESTIMATE algorithm was used to analyze the immune, stromal, and ESTIMATE scores. The differences in survival between the two groups were compared using the logarithmic rank test, and visualized using Kaplan-Meier.

The proportion of 28 immune cells in the tumor microenvironment is determined using the single-sample gene set enrichment analysis (ssGSEA) method.

### Statistical analysis

The statistical analysis was performed using Stata 15.0. It involved calculating the correlations between TLS and OS, RFS, PFS, and DFS. If P<0.05 and I^2^ >50%, it indicated high heterogeneity, and a random-effects model was applied. Otherwise, a fixed-effects model was used. Egger’s and Begg’s tests were employed to assess publication bias. If significant publication bias was detected, the trim and fill method was utilized to adjust the results. Additionally, a sensitivity analysis was conducted by systematically excluding individual studies in order to evaluate the robustness of the meta-analysis. P<0.05 was considered statistically significant.

## Results

### Characteristics of studies

**Figure 1:**
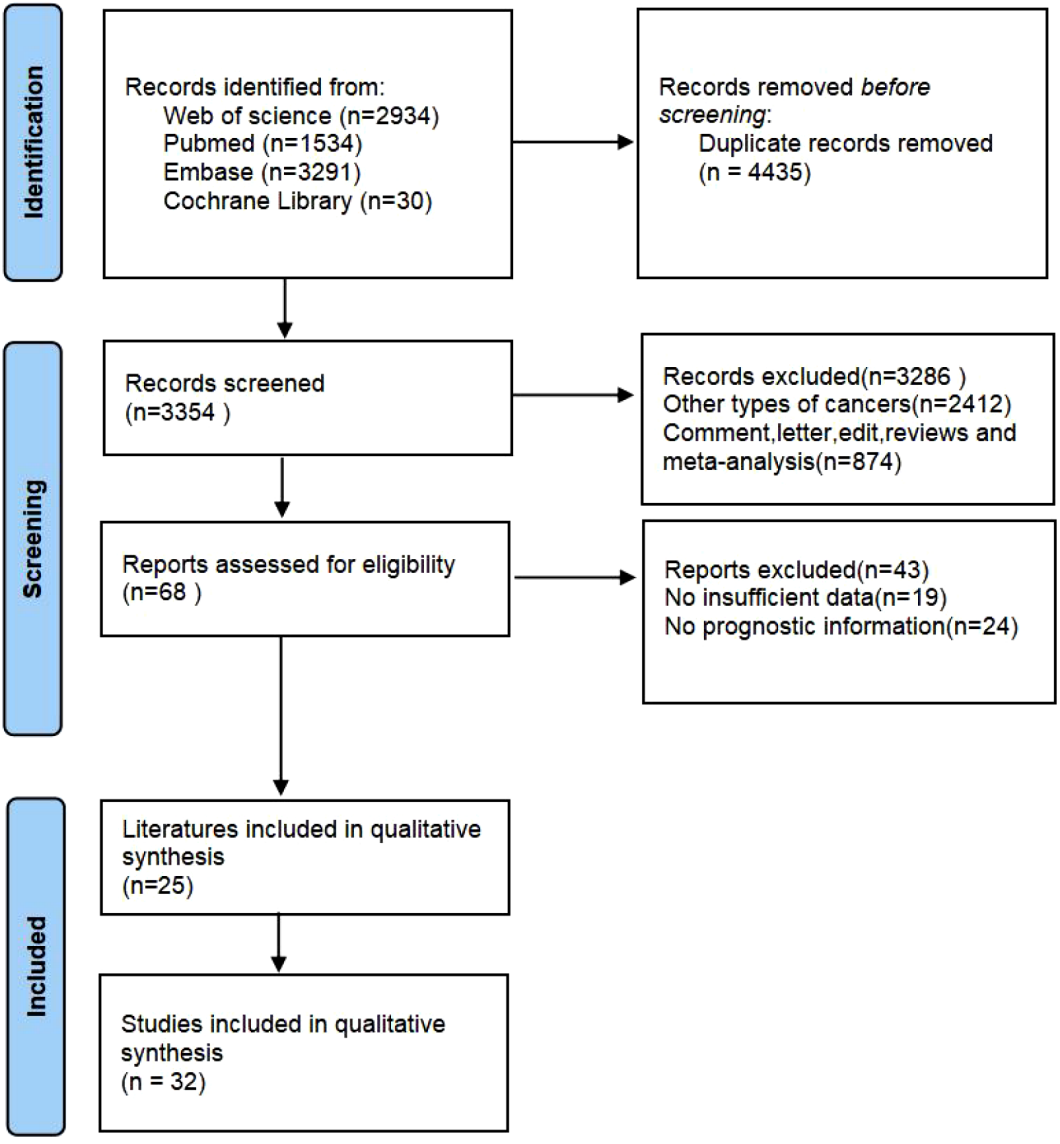
The flow diagram of identifying eligible studies.

After the initial search, we eliminated a total of 4435 duplicate articles. Then, we carefully read the titles and abstracts of the remaining articles and excluded 3286 of them. Subsequently, we obtained the full text of the remaining 64 articles and conducted a thorough evaluation. Finally, we selected and included 25 articles for our study (12–18, 23–40). These 25 articles encompassed 32 individual studies and involved a total of 5778 patients. The PRISMA flowchart depicting the entire selection process can be seen in the provided (**Figure 1**).

The included studies in this research are summarized in (**Table 1**), which consists of 8 studies on gastric cancer (GC), 6 studies on colorectal cancer (CRC), 7 studies on hepatocellular carcinoma (HCC), 4 studies on esophageal cancer (EC), 4 studies on pancreatic cancer (PC), and 3 studies on intrahepatic cholangiocarcinoma (ICC). Among these studies, 20 were conducted in China, 7 were from Japan, and 2 each were from Finland, the United States, Australia, and Germany. Moreover, 10 studies only provided overall survival (OS) data, 1 study reported only disease-free survival (DFS) data, 1 study solely focused on relapse-free survival (RFS) data, and another study presented progression-free survival (PFS) data exclusively. Interestingly, 11 studies provided both OS and RFS data, while 4 studies included both OS and DFS data, as well as OS and PFS data. The incorporated studies employed four cut-off criteria to designate TLS: namely, Presence, Density, Degree of maturation, and Maximum dimension. The NOS scores of the 32 studies ranged from 6 to 8, signifying an exceptional standard of the encompassed research. Every study embraced pertinent insights regarding TLS within malignant growths in this article.

**Table 1:** Characteristics of included studies.

Study	region	Sample size	Male/Female	Cancer types	Cut-off criteria	Follow-up time(months)	Survival analysis	NOS score
Cheng N.2021 (16)	China	846	585/261	GC	Presence	22.1 (1–99)	OS	8
Yu J.2022 (15)	China	118	82/36	GC	Density	(0-120)	OS,DFS	7
Kemi N.2023 (23)	Finland	583	425/296	GC	maximal diameter	28(1,432)	OS	8
Yin Y.2022(Training) (17)	China	148	131/17	GC	Degree of maturity	(0-60)	OS	8
Yin Y.2022(Validation) (17)	China	76	NR	GC	Degree of maturity	NR	OS	8
Mori Y.2021 (24)	Japan	261	182/92	GC	Density	(0-70)	OS	7
Mori Y.2022(ICIs) (25)	Japan	19	12/7	GC	Density	(0-27)	OS,PFS	7
Yamakoshi Y.2020 (12)	Japan	226	162/64	GC	Density	(0-90)	DFS	8
Ahmed A.2020 (18)	German	21	14/7	CRC	Density	(0-70)	OS	6
Zhan Z.2023 (26)	China	203	128/75	CRC	Density	50(0-70)	OS,DFS	8
Schweiger T.2016 (14)	Austria	57	33/24	CRC	Presence	(1-140)	OS,RFS	7
Karjula T.2023 (27)	Finland	67	34/33	CRC	Density, maximal diameter	40.2(5-233)	OS	7
Wang Q. 2022(Training) (40)	China	114	65/49	CRC	Presence	(0-60)	OS,RFS	8
Wang Q. 2022(Validation) (40)	China	60	37/23	CRC	Presence	(0-60)	OS,RFS	8
Wen S.2021 (28)	China	85	75/13	HCC	Density	44(0-60)	OS	8
Li H.2021(Training) (29)	China	240	202/38	HCC	Density, Presence	60.3(2.4-111.7)	OS,RFS	8
Li H.2021(Validation) (29)	China	120	99/21	HCC	Density, Presence	NR	OS,RFS	8
Li J.2022 (30)	China	150	125/25	HCC	Presence	(0-80)	OS,RFS	7
Li H.2020(Training) (31)	China	303	251/52	HCC	Presence	61.3(1.5-119.4)	OS,RFS	8
Li H.2020(Validation) (31)	China	159	132/27	HCC	Presence	NR	OS,RFS	8
Zhang T.2022 (32)	China	170	143/27	HCC	Density	(0-70)	OS,DFS	7
Hayashi Y.2023 (33)	Japan	316	255/61	EC	Density	(0-120)	OS,PFS	7
Hayashi Y.2023(ICIs) (33)	Japan	34	27/7	EC	Density	(0-41)	PFS	7
Deguchi S.2022 (35)	Japan	84	NR	EC	Presence	51(0-100)	RFS	7
Li R.2022(Training) (34)	China	122	102/20	EC	Density, Presence	(0-50)	OS,DFS	8
Zhang W.(Training) (13)	China	182	87/95	PC	Presence	39 (1.5-95)	OS,RFS	7
Zhang W.(Validation) (13)	China	125	60/65	PC	Presence	58 (10–96)	OS,RFS	7
Gunderson A.2021 (36)	America	63	37/26	PC	Density	(0–64)	OS	8
Tanaka T.2023 (37)	Japan	162	90/72	PC	Presence	26 (1–122)	OS	8
Shang T.2023 (38)	China	471	160/311	ICC	Density	(0–80)	OS,PFS	8
Shang T.2023(ICIs) (38)	China	100	NR	ICC	Density	(0–50)	OS,PFS	8
Zhang P.2022 (39)	China	93	53/40	ICC	Degree of maturity, Presence	(0-108)	OS,RFS	8

GC Gastric cancer, CRC Colorectal cancer, EC Esophageal cancer, HCC Hepatocellular carcinoma, PC Pancreatic cancer, ICC Cholangiocarcinoma.

Follow-up time(months):medians(ranges).

NR, Not available.

TLS is divided into TLS-high and TLS-low based on different cut-off criteria. Among the 31 studies included in this paper, different cut-off criteria correspond to different HR. In the subsequent investigation of the relationship between TLS and OS, RFS, PFS, and DFS, we have established inclusion criteria. When a study includes two different TLS cut-off criteria, we prioritize the HR corresponding to Density, Degree of maturity, or Maximal diameter. If a study simultaneously uses Density and Degree of maturity or Density and Maximal diameter as the grading methods for TLS, we select the HR corresponding to Density.

### TLS and OS

**Figure 2:**
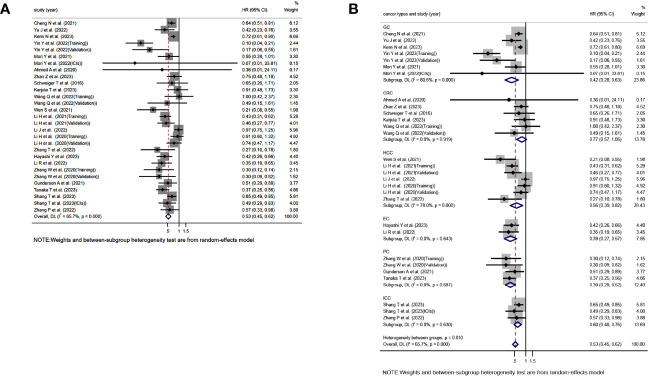
Forest plot showing the relationship between TLS and OS in GI cancers. **(A)** OS; **(B)** OS subgroup analysis for different cancer types.HR, hazard ratio; CL, confidence interval.

Based on a comprehensive analysis of 29 studies evaluating the association between TLS and OS in GI cancer, it was found that TLS-high is significantly correlated with longer OS(HR=0.525, 95%CI:0.447-0.616, P<0.001)(**Figure 2A**). However, it should be noted that this analysis showed significant heterogeneity, and a random effects model was used to account for this(*I* ^2 =^ 65.7%, P<0.001)(**Figure 2A**). Moreover, subgroup analysis based on different cancer types revealed that TLS-high is closely associated with extended OS in GC(HR=0.422, 95%CI:0.283-0.627,P<0.001), HCC(HR=0.532, 95%CI:0.391-0.726,P=0.003), EC(HR=0.393, 95%CI:0.271-0.570,P<0.001), PC(HR=0.390, 95%CI:0.290-0.525,P<0.001), and ICC(HR=0.493, 95%CI:0.421-0.577,P<0.001), while no significant relationship was found between TLS and OS in CRC(HR=0.775, 95%CI:0.570-1.053,P=0.103) (**Figure 2B**). Interestingly, significant heterogeneity was observed in GC(*I* ^2 =^ 80.6%,P<0.001) and HCC(*I* ^2 =^ 78.0%,P<0.001) (**Figure 2B**).

### TLS and RFS, DFS, PFS

**Figure 3:**
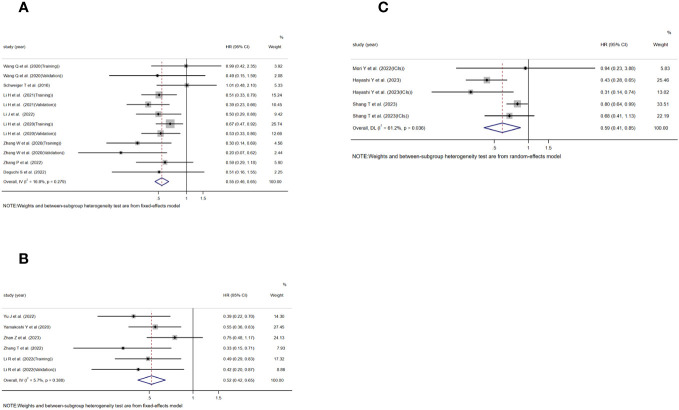
Forest plot showing the relationship between TLS and RFS, DFS, PFS in GI cancers. **(A)** RFS; **(B)** DFS; **(C)** PFS.

Moving on to the assessment of TLS in relation to RFS, DFS, and PFS, 12, 6, and 5 studies were included, respectively. The analysis showed a significant association between TLS-high and extended RFS(HR=0.546, 95%CI:0.461-0.647, P<0.001)(**Figure 3A**), DFS(HR=0.519, 95%CI:0.417-0.646, P<0.001)(**Figure 3B**), and PFS(HR=0.588, 95%CI:0.406-0.852, P=0.005)(**Figure 3C**). Notably, the analysis of RFS(*I* ^2 =^ 16.8%, P=0.279)(**Figure 3A**)and DFS *I* ^2 =^ 5.7%, P=0.380)(**Figure 3B**) did not exhibit significant heterogeneity, and a fixed effects model was used, whereas significant heterogeneity was observed in the analysis of PFS[*I* ^2 =^ 61.2%, P=0.036)](**Figure 3C**), requiring the use of a random effects model.

### TLS and ICIs

**Figure 4:**
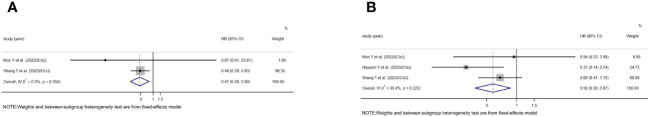
Forest plot showing the relationship between TLS and OS, PFS in GI cancers undergoing treatment with immune ICIs. **(A)** OS; **(B)** PFS.

In addition, two studies assessing the relationship between TLS and OS in the context of using ICIs for treatment were included, as well as three studies evaluating the relationship between TLS and PFS. The study conducted by Mori Y et al. and Hayashi Y et al. included patients who received treatment with anti-PD-1 monoclonal antibodies. On the other hand, the study conducted by Shang T et al. did not explicitly specify the type of ICIs used in their research. The results consistently showed a significant association between TLS-positive and extended OS[HR=0.475, 95%CI:0.282-0.799, P=0.005)](**Figure 4A**) and PFS[HR=0.576, 95%CI:0.381-0.871, P=0.009)](**Figure 4B**). No significant heterogeneity was found in any of these studies, and a fixed effects model was applied(*I* ^2 =^ 0.0%, P=0.352)(**Figure 4A**), (*I* ^2 =^ 33.4%, P=0.223)(**Figure 4B**).

### TLS and cut-off criteria

Notably, in previous studies examining the relationship between TLS and OS, significant heterogeneity was still observed in the GC and HCC subgroups. This may be attributed to the use of different TLS cut-off criteria in some studies, resulting in different conclusions. Therefore, to ascertain the potential impact of cut-off criteria on the evaluation of TLS prognosis, studies using different criteria such as Presence, Density, Degree of maturity, and Maximal diameter were included. However, due to the limited number of included studies, further investigation on the impact of cut-off criteria on DFS and PFS was not possible. Firstly, we included 15 studies that used presence as a cut-off criterion to study the relationship between TLS and OS. The evaluation showed a significant correlation between TLS-high and prolonged OS in the included studies(HR=0.590, 95%CI:0.474-0.733, P<0.001)(**Figure S1A**). However, there was significant heterogeneity observed, so a random-effects model was used(*I* ^2 =^ 62.7%, P=0.001)(**Figure S1A**). Subgroup analysis based on cancer types revealed no significant association between TLS and OS in HCC(HR=0.850, 95%CI:0.721-1.002, P=0.053)and CRC(HR=0.731, 95%CI:0.417-1.282, P=0.272), while a significant correlation was found in PC (HR=0.351, 95%CI:0.248-0.498, P<0.001)and other tumors(HR=0.629, 95%CI:0.508-0.778, P<0.001)(**Figure S1B**). Next, we included 15 studies that used density as a cut-off criterion to study the relationship between TLS and OS. The evaluation showed a significant correlation between TLS positivity and prolonged OS in the included studies(HR= 0.516, 95%CI:0.450-0.591, P<0.001)(**Figure S1C**). No significant heterogeneity was found, so a fixed-effects model was used(*I* ^2 =^ 23.3%, P=0.195)(**Figure S1C**). Subgroup analysis based on cancer types showed a close correlation between TLS-high and OS prolongation in GC(HR=0.466, 95%CI:0.302-0.719,P<0.001), HCC(HR=0.401, 95%CI:0.307-0.524, P<0.001),EC(HR=0.393, 95%CI:0.271-0.570,P<0.001),PC(HR=0.510, 95%CI:0.291-0.893,P=0.019) and ICC(HR=0.612, 95%CI:0.479-0.781,P<0.001) (**Figure S1D**). However, no significant relationship was observed in CRC(HR=0.794, 95%CI:0.550-1.145,P=0.217) (**Figure S1D**). Furthermore, after including 3 studies that used maturity as a criteria to study the relationship between TLS and OS, we found a similar significant correlation between TLS-high and prolonged OS(HR=0.222, 95%CI:0.068-0.0730, P=0.013)(**Figure S1E**). Due to significant heterogeneity observed in the studies, a random-effects model was used(*I* ^2 =^ 84.7%, P=0.001)(**Figure S1E**). Moving on to the relationship between TLS and RFS, we included 10 studies that used presence as a cut-off criterion. We found a significant correlation between TLS and prolonged RFS in these studies(HR=0.591, 95%CI:0.495-0.705, P<0.001)(**Figure S2A**). No significant heterogeneity was observed, so a fixed-effects model was used [(*I* ^2 =^ 33.5%, P<0.122](**Figure S2A**). Subgroup analysis based on cancer types revealed no significant association between TLS and RFS in CRC(HR=0.874, 95%CI:0.527-1.450, P=0.602)(**Figure S2B**), while a significant correlation was found in HCC(HR=0.638, 95%CI:0.518-0.787, P<0.001), PC(HR=0.260, 95%CI:0.137-0.496, P<0.001), and other tumors HR=0.365, 95%CI:0.200-0.665, P=0.001)(**Figure S2B**). In the 2 studies that evaluated the relationship between TLS and RFS using density as a cut-off criteria, we found a significant correlation between TLS and RFS(HR=0.457, 95%CI:0.327-0.640, P<0.001)(**Figure S2C**). No significant heterogeneity was found in these studies, so a fixed-effects model was used(*I* ^2 =^ 0.0%, P=0.442)(**Figure S2C**).

We found that different studies have used various criteria such as ROC curves, medians, and other ambiguous methods to divide the Density of TLS into two parts, namely TLS-high and TLS-low, when using Density as the cut-off for TLS. We conducted a meta-regression to determine if different criteria would affect the predictive value of TLS. The different criteria used to divide Density can affect the predictive value of TLS(P=0.023). Further sub-analysis demonstrates that TLS has a significant correlation with OS across various criteria (**Figure S3**).

### Sensitivity analysis and publication bias

To assess sensitivity, we employed the leave-one-out method for statistical analysis. After systematically excluding each individual study, the overall (HR) for OS, RFS, DFS, and PFS did not show any significant changes, indicating the stability and reliability of our findings (**Figure S4**).

Next, we employed a funnel plot (**Figure S5**), Begg’s test (**Figure S6**), and Egger’s test (**Figure S6**) to evaluate publication bias in the included studies. We found evidence of publication bias in OS(Begg’s test:P=0.063, Egger’s test: P=0.001)(**Figures S6 A, H**). However, no publication bias was observed in RFS(Begg’s test:P=0.732, Egger’s test:P=0.430)(**Figures S6 B, I**), DFS(Begg’s test:P=0.060, Egger’s test:P=0.063)(**Figures S6 C, J**), and PFS(Begg’s test:P=1.000, Egger’s test: P=0.364)(**Figures S6 D, K**). Further analysis of the cut-off criteria for TLS revealed publication bias in OS (Begg’s test:P=0.322, Egger’s test:P=0.033)in the “Density group”(**Figures S6 F, M**). There was no publication bias in OS(Begg’s test:P=0.235, Egger’s test:P=0.064) in the “Presence group”(**Figures S6 E, L**), and RFS(Begg’s test:P=0.210, Egger’s test:P=0.125) in the “Presence group”(**Figures S6 F, N**). All remaining studies from the subgroups mentioned above were included in our analysis. Subsequently, we applied the trim and fill method to fill in the missing data from studies that had zero items missing. This approach ensured that our results remained robust and reliable.

### TLS and bioinformatics analysis

**Figure 5:**
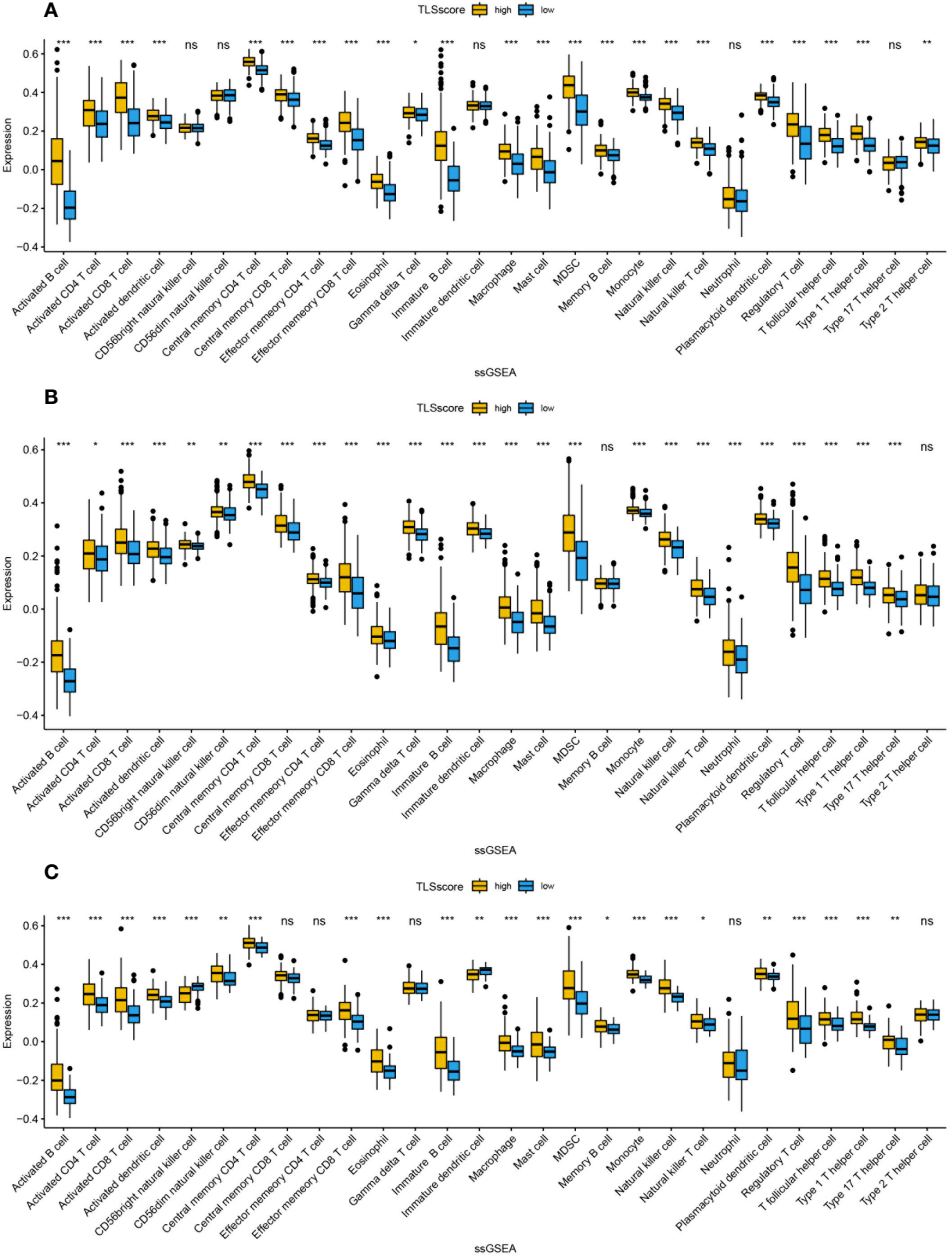
The abundance of infiltrating cells in each tumor microenvironment based on ssGSEA analysis of two groups of TLSscore. **(A)** GC, **(B)** CRC, **(C)** EC. *: P<0.05, **: P<0.01, ***: P<0.001, ns: P≥0.05 non-significant.

**Figure 6:**
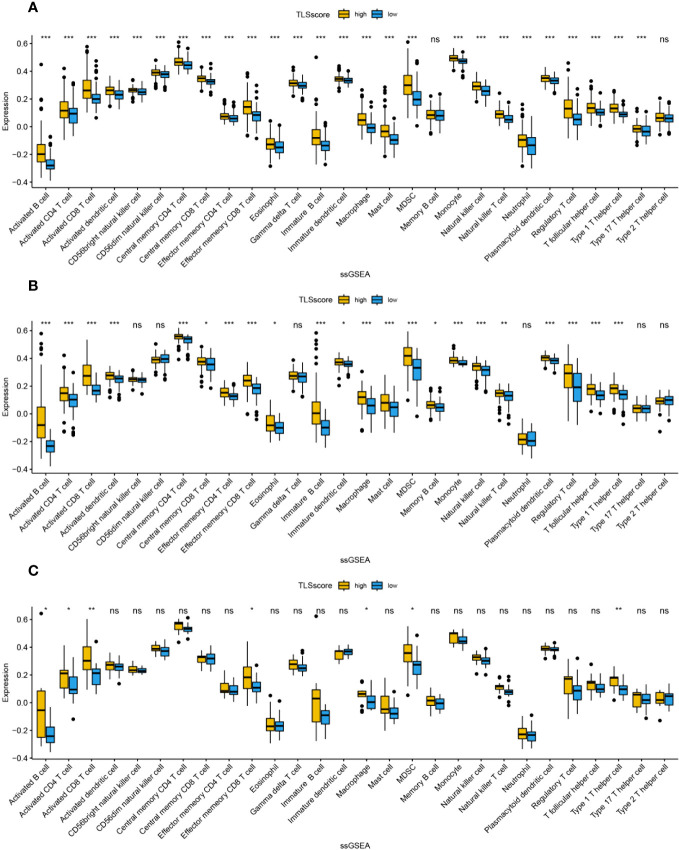
The abundance of infiltrating cells in each tumor microenvironment based on ssGSEA analysis of two groups of TLSscore. **(A)** HCC, **(B)** PC, **(C)** ICC. *: P<0.05, **: P<0.01, ***: P<0.001, ns: P≥0.05 non-significant.

We studied the relationship between TLSscore and the immune microenvironment. In the ESTIMATE algorithm, patients in the high TLSscore group showed higher immune, stromal, and ESTIMATE scores (**Figure S7**). Single-sample gene set enrichment analysis (ssGSEA) revealed a significantly higher degree of infiltration of various immune-related cells in the TLSscore high group compared to the TLSscore low group (**Figures 5**, **6**). Additionally, we further investigated the relationship between TLSscore and prognosis. In HCC, we found a significant improvement in overall survival (OS) associated with TLSscore high, while no significant association between TLSscore and OS was found in other gastrointestinal cancers (**Figure S8**).

## Discussion

Compared to TLS-low tumors, TLS-high tumors exhibit overexpression of a set of genes that promote T cell activation, T helper 1 (TH-1) cell skewing, T cell chemotaxis, and T cell cytotoxicity (41). Moreover, the unique spatial structure of TLS facilitates the presentation of antigen peptides by mature dendritic cells (DC) and potential B cells in the T cell zone, activating them to generate a response against tumor cells presenting the same antigen (9, 42). The Phase 2 PEMBROSARC trial cohort has provided evidence that TLS serves as a novel biological biomarker improving treatment selection for patients with advanced soft tissue sarcoma (STS) undergoing pembrolizumab therapy (43). This reflects the important predictive role of TLS in tumor immunotherapy response. Some studies suggest that the presence of TLS may be associated with the activation of anti-tumor immune responses and further contribute to the anti-tumor effects (44–48). There is a significant variation in clinical prognosis among patients with GI cancer, even within the same TMN stage. Additionally, there is a scarcity of prognostic markers for cancer immunotherapy and they are often difficult to meet clinical needs (49). Therefore, the search for biomarkers that can be used for early detection and prognosis assessment in cancer is of urgent importance.

The purpose of this study is to investigate the relationship between TLS (Tertiary Lymphoid Structures) and prognosis of GI (Gastrointestinal) cancer (50). While previous literature has explored the association between TLS and prognosis of solid tumors, there is still relatively limited information regarding GI cancer. In this study, we updated the information on GI cancer and conducted subgroup analysis to clarify the relationship between TLS and overall survival (OS), progression-free survival (PFS), disease-free survival (DFS), and recurrence-free survival (RFS). The results indicated that TLS was significantly associated with prolonged OS, PFS, DFS, and RFS. However, in the subgroup analysis specifically focusing on colorectal cancer, TLS was not significantly associated with OS. A possible explanation may be due to the presence of GALT tissue or Peyer’s patches that preexist TLS and are considered as TLS due the inclusion of these genes in those normal lymphoid tissues (51, 52). Wang Q and colleagues suggested that the higher proportion of regulatory T cells (Treg) within TLS in tumors might be one of the mechanisms that undermine its prognostic value in CRC (40). Additionally, TLS was significantly associated with OS and PFS in patients receiving immunotherapy. Furthermore, we investigated whether different cut-off criteria would affect the predictive value of TLS for prognosis in GI cancer. In hepatocellular carcinoma (HCC), TLS positivity did not improve patient OS when using “Presence” as the cut-off criteria, whereas it was significantly associated with prolonged OS when using “Density” as the cut-off criteria. This suggests that different cut-off criteria can influence the predictive value of TLS for prognosis in GI cancer, especially in HCC where “Density” is more suitable as the cut-off criteria for TLS. Moreover, in CRC, three studies using “Density” as the cut-off criteria showed no significant association between TLS and OS, and three studies using “Presence” as the cut-off criteria also did not yield meaningful results. Wang Q and colleagues found that TLS density in the surrounding normal tissue could predict the prognosis of CRC patients (40). Additionally, Yamaguchi K and colleagues’ research indicated that a T helper (Th) cell-dominant composition within TLS was an independent risk factor for postoperative recurrence of CRC (53). Li Q and his team have linked the presence of TLS with the density of tumor-infiltrating lymphocytes, and discovered that this combination acts as a prognostic biomarker for oral cancer. The results of their research, as depicted by the Receiver Operating Characteristic (ROC) curve, demonstrated that this combined marker exhibits a remarkably high predictive accuracy for 5-year OS (54). These novel detection methods have the potential to expand the prognostic value of TLS and make TLS a biological marker for CRC prognosis.

In addition, due to significant heterogeneity among TLS identification methods, we further investigated the relationship between the immune microenvironment and TLS in gastrointestinal cancer in the TCGA database using the method proposed by Cabrita et al. to quantify TLSscore based on 9 TLS-related genes. We found that patients in the TLSscore high group had higher levels of immune cell infiltration compared to those in the TLSscore low group. Recent studies have shown that the formation of TLS is mediated by certain pro-inflammatory cytokines and TNF receptor family members, and involves the participation of fibroblasts, perivascular myofibroblasts, and stromal cells (55). However, in gastrointestinal cancer, a large number of patients have low tumor mutation burden and lack immune cell infiltration, making their tumor microenvironment “cold” and resulting in poor response to emerging therapies targeting the tumor immune microenvironment, such as immunotherapy (56, 57). Hooren et al. discovered the formation of TLS during the process of transforming the solid tumor immune microenvironment from “cold” to “hot” using a CD40 agonist, and TLS was found to be correlated with increased infiltration of T cells (58). We speculate that appropriate immune cell infiltration may be associated with the formation of TLS, and the presence of TLS also promotes the infiltration of local immune cells. Numerous studies have shown a significant prolongation of survival time with high density of immune cell infiltration (59–61), which partially explains the improvement in prognosis with TLS. However, our survival analysis based on the TCGA database showed that only liver cancer exhibited a predictive value of TLSscore for prognosis in gastrointestinal cancer, which suggests that we need to consider the applicability of the “9-gene method” in gastrointestinal cancer and further investigate the consistency of different methods such as IHC, HE, and gene markers in TLS evaluation in gastrointestinal cancer in real-world studies. Jiang et al. divided gastric cancer patients in the GSE84437 and TCGA cohorts into two groups based on unsupervised clustering analysis of 39 TLS-related genes, and significant differences in prognosis and immune scores were observed between the two groups (62). We look forward to future validation of the accuracy of the TLS biomarker assessment proposed by them in the real world.

There are several limitations in this meta-analysis. Firstly, some articles did not provide sufficient prognostic data, and some survival statistics data calculated from survival curves using Engauge Digitizer may have biases. Secondly, the majority of included studies were from Asia, which may result in a publication bias to some extent. Additionally, there were fewer studies included in some subgroup analyses, especially in the subgroup of immunotherapy, with only 3 immunotherapy studies included in the analysis and a relatively small sample size (153 cases in total), which may affect the evaluation of the role of TLS in prognosis. Lastly, this meta-analysis only included data related to intratumoral TLS, which cannot fully reflect its predictive role in prognosis.

## Conclusion

Despite its limitations, we can conclude that TLS can serve as an excellent prognostic factor for GI cancer, and appropriate cut-off criteria should be selected for different cancer subtypes. In CRC, the focus can be on TLS in the surrounding normal tissue of the tumor or in combination with other predictive indicators to serve as prognostic markers. Furthermore, high-quality and multicenter clinical studies, especially in immunotherapy cohorts, are needed to further elucidate the impact of TLS on the survival outcomes of GI cancer.

## Data availability statement

The original contributions presented in the study are included in the article/**Supplementary Material**. Further inquiries can be directed to the corresponding authors.

## Author contributions

AY: Writing – original draft. MC: Data curation, Writing – original draft. KZ: Data curation, Methodology, Writing – original draft. YY: Data curation, Formal Analysis, Writing – original draft. LM: Data curation, Formal Analysis, Writing – original draft. XZ: Data curation, Writing – original draft. YZ: Writing – original draft. XM: Writing – original draft. ZF: Writing – original draft. ZH: Writing – review & editing, Conceptualization. HW: Writing – review & editing.

## Supplementary material

Supplementary Figure 1 Forest plot showing the relationship between TLS and OS in GI cancers under different cut-off criteria. **(A)** OS when presence is used as a cut-off criterion; **(B)** OS subgroup analysis for different cancer types when presence is used as a cut-off criterion. **(C)** OS when density is used as a cut-off criterion. **(D)** OS subgroup analysis for different cancer types when density is used as a cut-off criterion. **(E)** OS when maturity is used as a cut-off criterion.

Supplementary Figure 2 Forest plot showing the relationship between TLS and RFS in GI cancers under different cut-off criteria. **(A)** RFS when presence is used as a cut-off criterion; **(B)** RFS subgroup analysis for different cancer types when presence is used as a cut-off criterion. **(C)** RFS when density is used as a cut-off criterion.

Supplementary Figure 3 Forest plot showing the relationship between TLS and OS in GI cancers under different criteria.

Supplementary Figure 4 Sensitivity analysis. **(A)**OS; **(B)**RFS; **(C)**DFS; **(D)**PFS; **(E)** OS when presence is used as a cut-off criterion;**(F)** OS when density is used as a cut-off criterion; **(G)** RFS when presence is used as a cut-off criterion.

Supplementary Figure 5 Funnel plots for detecting publication bias in terms of survival data. **(A)** OS; **(B)** RFS; **(C)** DFS; **(D)** PFS; **(E) **OS when presence is used as a cut-off criterion; **(F)** OS when density is used as a cut-off criterion; **(G)** RFS when presence is used as a cut-off criterion.

Supplementary Figure 6 Begg’s and Egger’s funnel plots for detecting publication bias in terms of survival data. **(A)** Begg’s test of OS;**(B)** Begg’s test of RFS; **(C)** Begg’s test of DFS; **(D)** Begg’s test of PFS; **(E)** Begg’s test of OS when presence is used as a cut-off criterion; **(F)** Begg’s test of OS when density is used as a cut-off criterion; **(G)**Begg’s test of RFS when presence is used as a cut-off criterion; **(H)** Egger’s test of OS; **(I)** Egger’s test of RFS; **(J)** Egger’s test of DFS; **(K)** Egger’s test of PFS; **(L)** Egger’s test of OS when presence is used as a cut-off criterion;**(M)** Egger’s test of OS when density is used as a cut-off criterion; **(N)** Egger’s test of RFS when presence is used as a cut-off criterion.

Supplementary Figure 7 Two groups of TLSscore immunity, matrix, and ESTIMATE scores based on the analysis using the ESTIMATE algorithm. **(A)** GC, **(B)** CRC, **(C)** EC, **(D)** HCC, **(E)** PC, **(F)** ICC.

Supplementary Figure 8 Survival analysis of Gl cancers based on TLSscore grouping. **(A)** GC, **(B)** CRC, **(C)** EC, **(D)** HCC, **(E)** PC, **(F)** ICC.
