# Supplementary figures and images for: The prognostic value of the tertiary lymphoid structure in gastrointestinal cancers
Source: Front Immunol. 2023 Oct 6;14:1256355. doi: 10.3389/fimmu.2023.1256355 (PMC10590053; doi:10.3389/fimmu.2023.1256355)

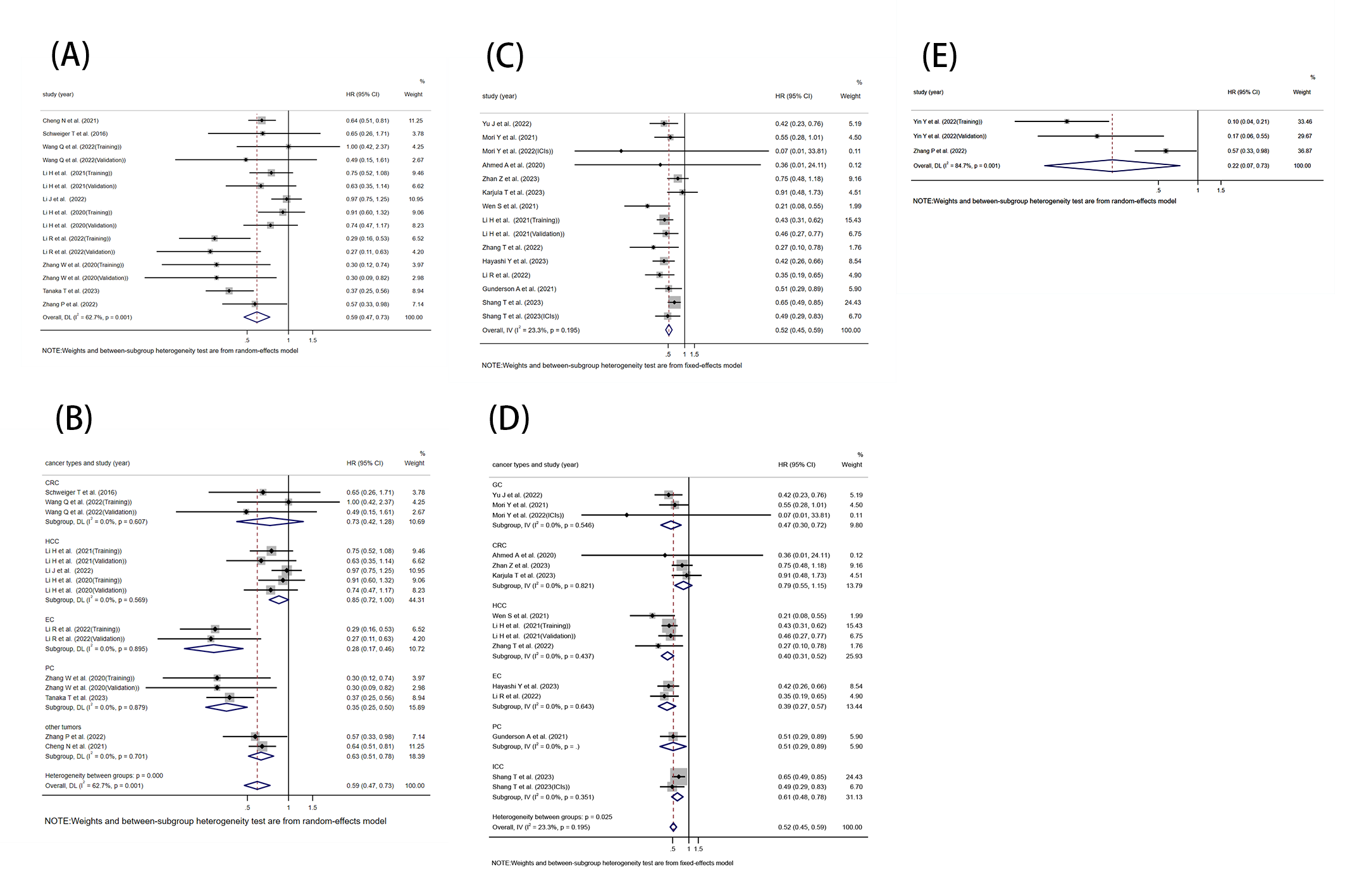

Supplement: Supplementary Figure 1 — Forest plot showing the relationship between TLS and OS in GI cancers under different cut-off criteria. (A) OS when presence is used as a cut-off criterion; (B) OS subgroup analysis for different cancer types when presence is used as a cut-off criterion. (C) OS when density is used as a cut-off criterion. (D) OS subgroup analysis for different cancer types when density is used as a cut-off criterion. (E) OS when maturity is used as a cut-off criterion. [file Image_1.tif]

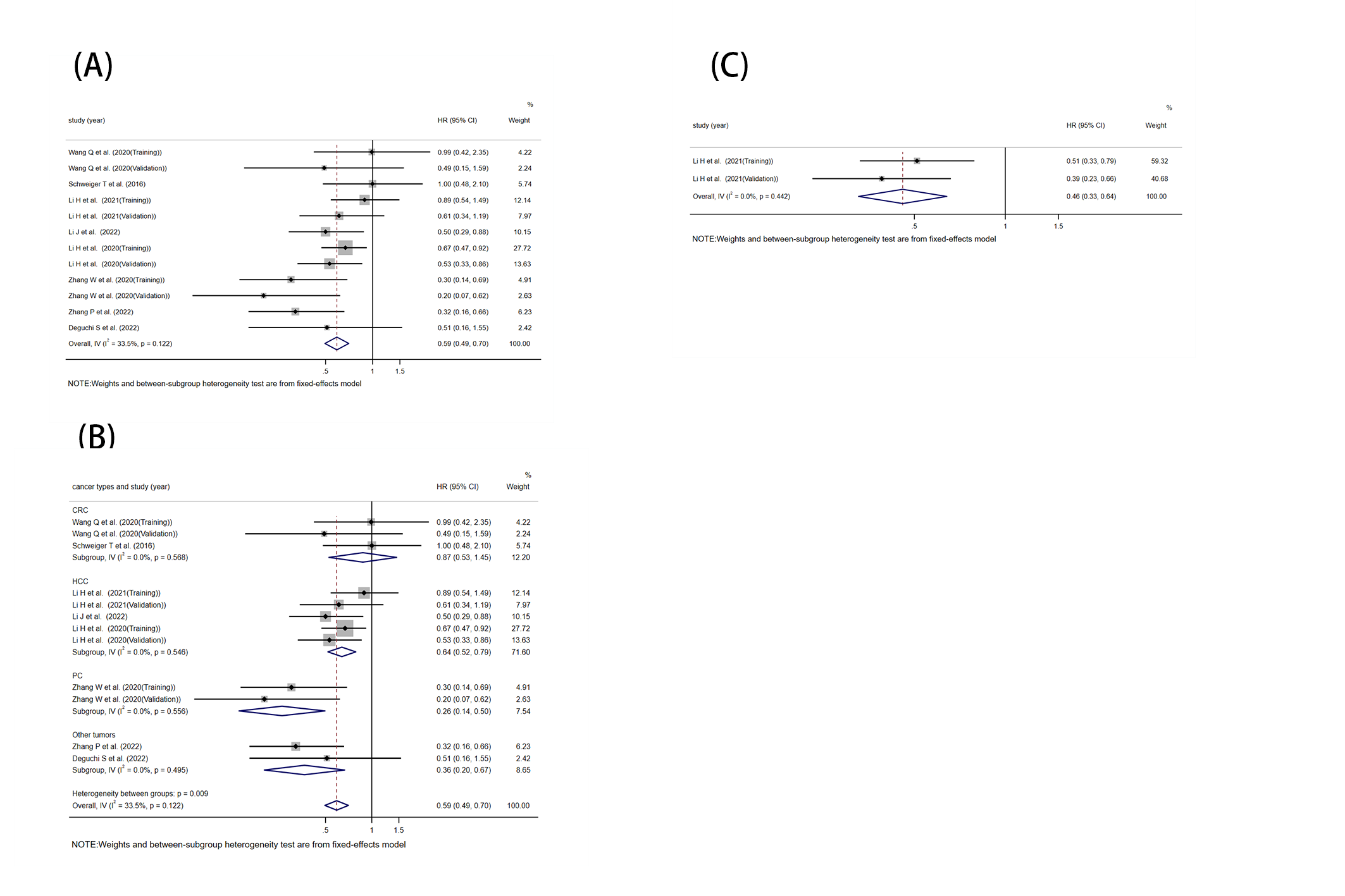

Supplement: Supplementary Figure 2 — Forest plot showing the relationship between TLS and RFS in GI cancers under different cut-off criteria. (A) RFS when presence is used as a cut-off criterion; (B) RFS subgroup analysis for different cancer types when presence is used as a cut-off criterion. (C) RFS when density is used as a cut-off criterion. [file Image_2.tif]

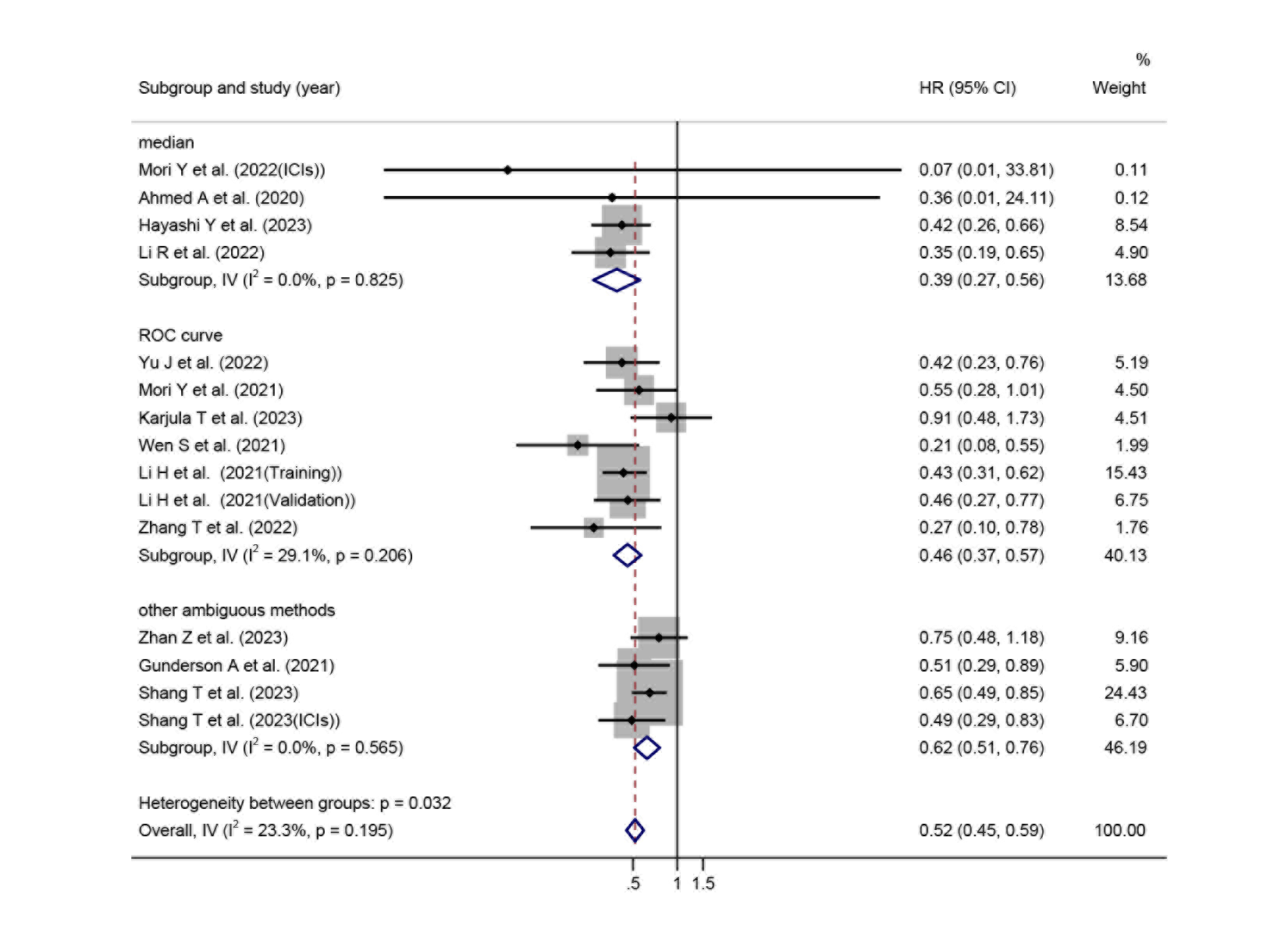

Supplement: Supplementary Figure 3 — Forest plot showing the relationship between TLS and OS in GI cancers under different criteria. [file Image_3.tif]

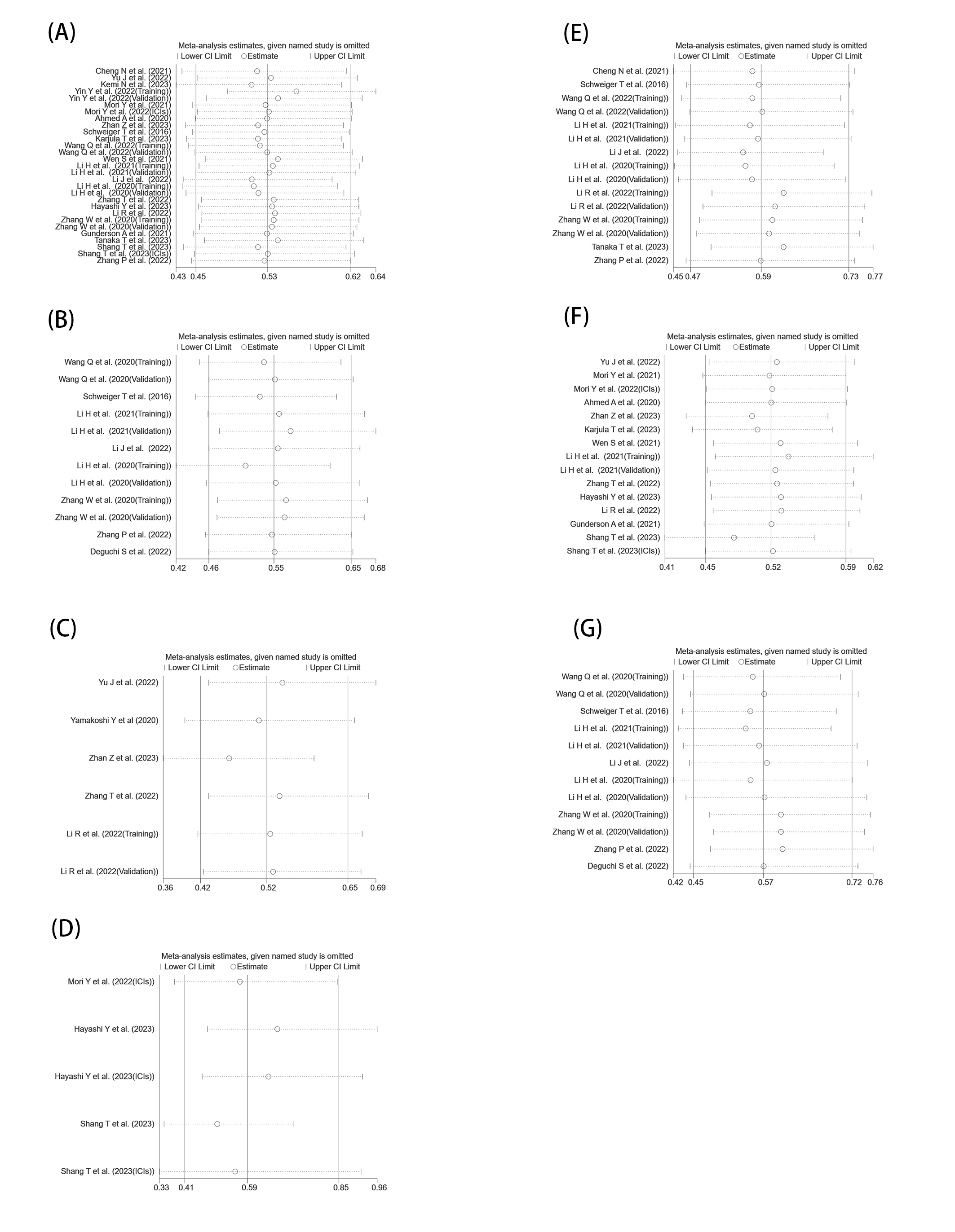

Supplement: Supplementary Figure 4 — Sensitivity analysis. (A)OS; (B)RFS; (C)DFS; (D)PFS; (E) OS when presence is used as a cut-off criterion;(F) OS when density is used as a cut-off criterion; (G) RFS when presence is used as a cut-off criterion. [file Image_4.tif]

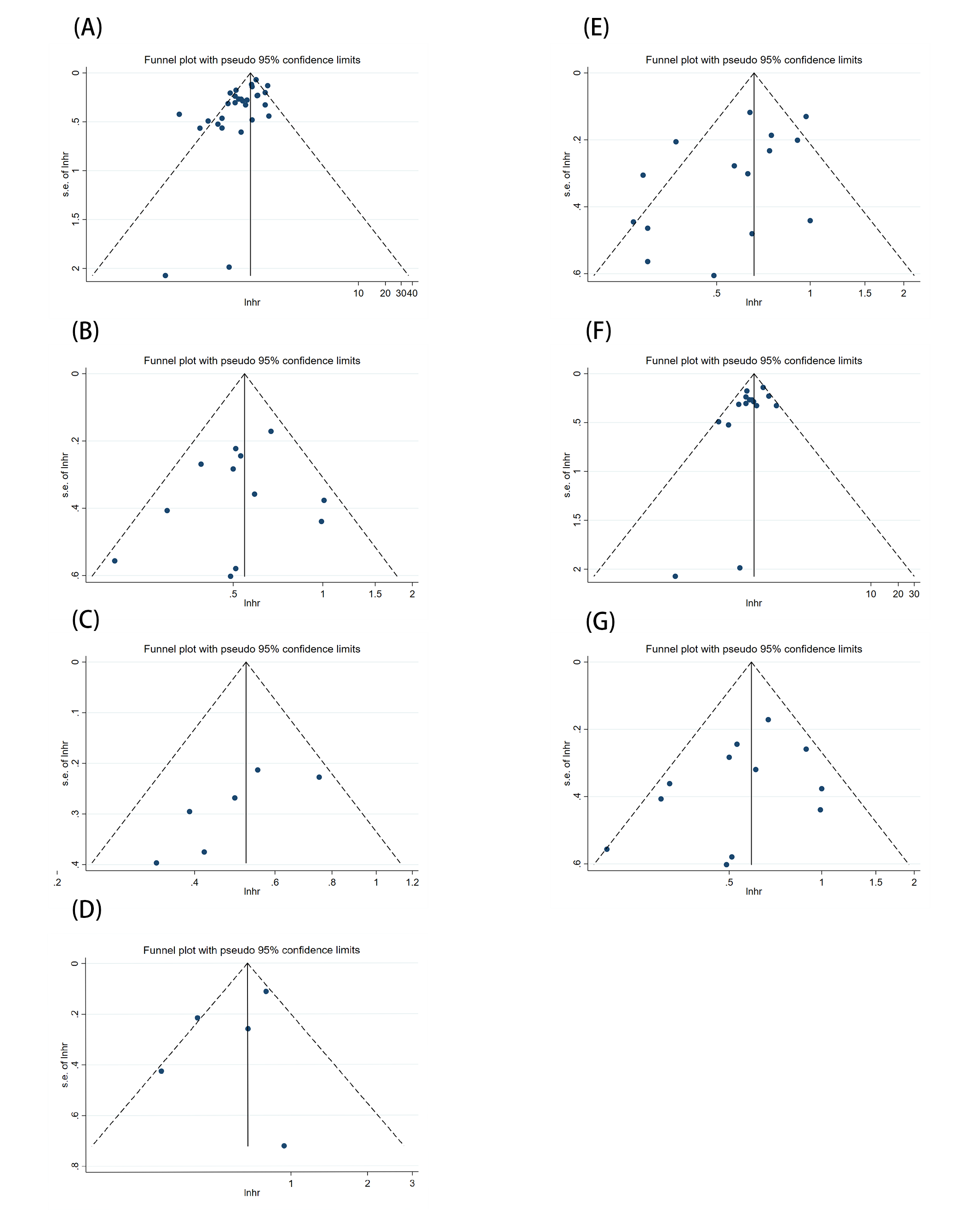

Supplement: Supplementary Figure 5 — Funnel plots for detecting publication bias in terms of survival data. (A) OS; (B) RFS; (C) DFS; (D) PFS; (E) OS when presence is used as a cut-off criterion; (F) OS when density is used as a cut-off criterion; (G) RFS when presence is used as a cut-off criterion. [file Image_5.tif]

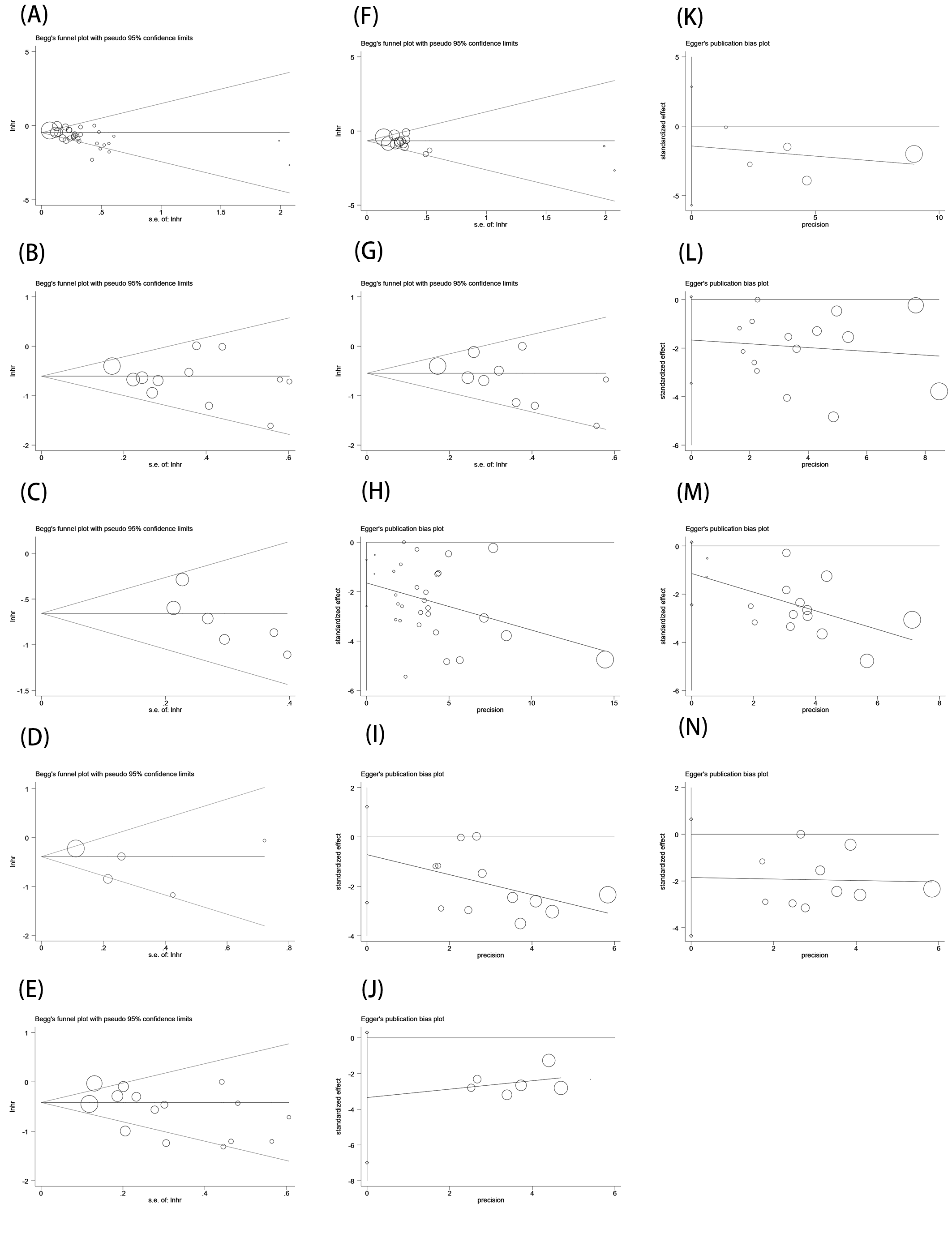

Supplement: Supplementary Figure 6 — Begg’s and Egger’s funnel plots for detecting publication bias in terms of survival data. (A) Begg’s test of OS;(B) Begg’s test of RFS; (C) Begg’s test of DFS; (D) Begg’s test of PFS; (E) Begg’s test of OS when presence is used as a cut-off criterion; (F) Begg’s test of OS when density is used as a cut-off criterion; (G)Begg’s test of RFS when presence is used as a cut-off criterion; (H) Egger’s test of OS; (I) Egger’s test of RFS; (J) Egger’s test of DFS; (K) Egger’s test of PFS; (L) Egger’s test of OS when presence is used as a cut-off criterion;(M) Egger’s test of OS when density is used as a cut-off criterion; (N) Egger’s test of RFS when presence is used as a cut-off criterion. [file Image_6.tif]

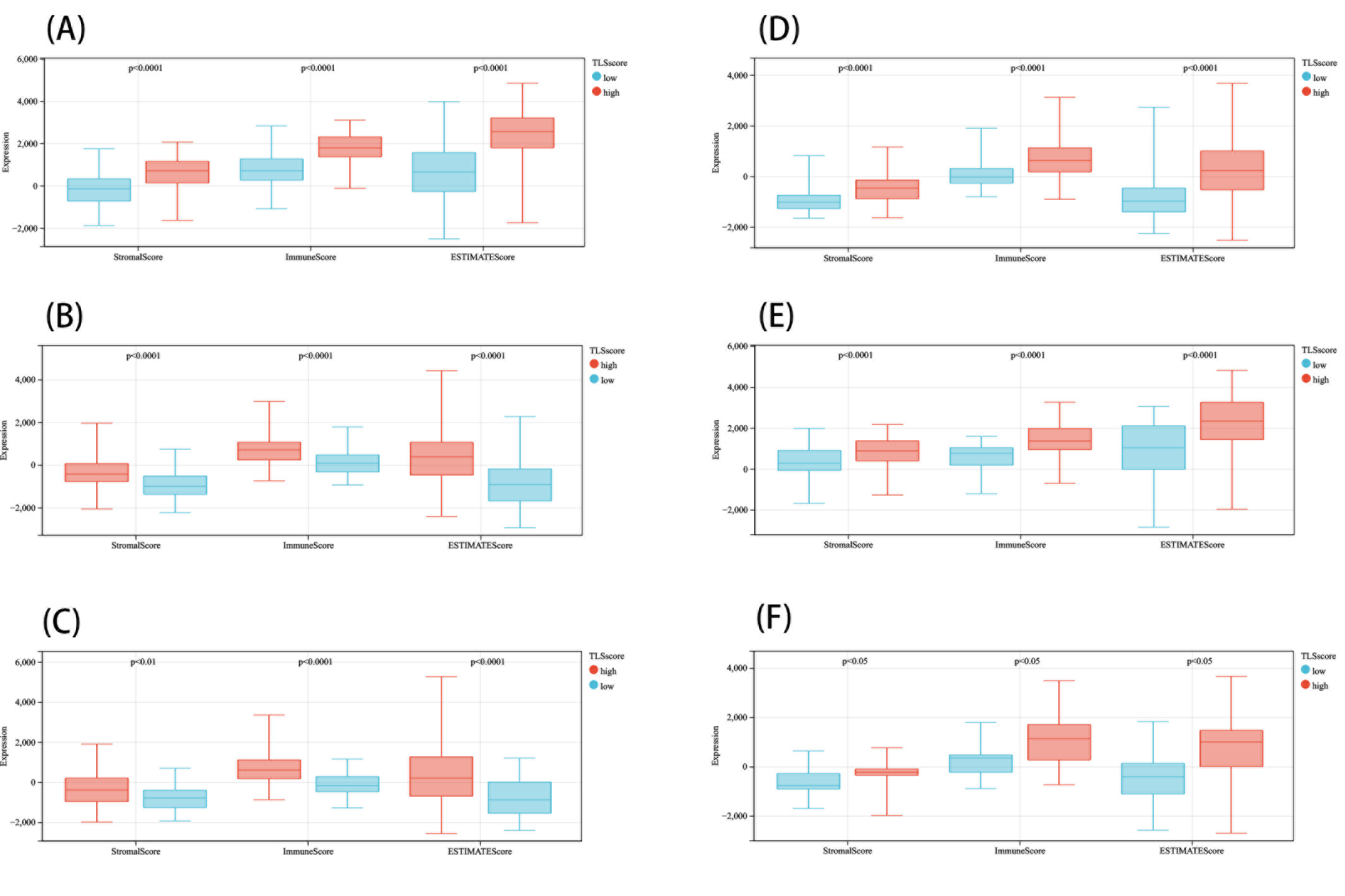

Supplement: Supplementary Figure 7 — Two groups of TLSscore immunity, matrix, and ESTIMATE scores based on the analysis using the ESTIMATE algorithm. (A) GC, (B) CRC, (C) EC, (D) HCC, (E) PC, (F) ICC. [file Image_7.tif]

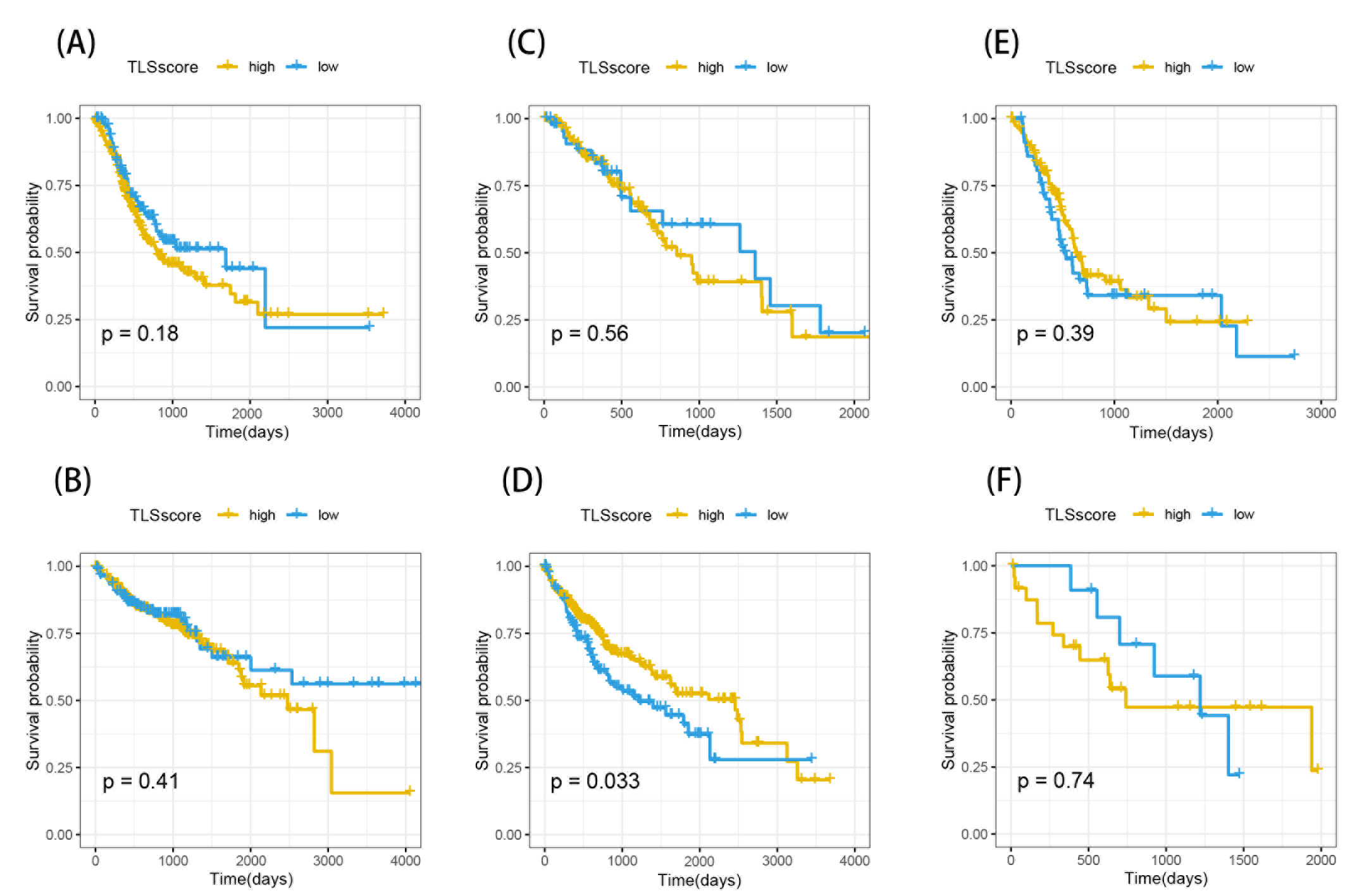

Supplement: Supplementary Figure 8 — Survival analysis of Gl cancers based on TLSscore grouping. (A) GC, (B) CRC, (C) EC, (D) HCC, (E) PC, (F) ICC. [file Image_8.tif]
